# Tumor Immune Microenvironment Heterogeneity at the Invasion Front and Tumor Center in Oral Squamous Cell Carcinoma as a Perspective of Managing This Cancer Entity

**DOI:** 10.3390/jcm12041704

**Published:** 2023-02-20

**Authors:** Andreas Mamilos, Alexander Lein, Lina Winter, Tobias Ettl, Julian Künzel, Torsten E. Reichert, Gerrit Spanier, Christoph Brochhausen

**Affiliations:** 1 Institute of Pathology, University of Regensburg, 93053 Regensburg, Germany; 2 Central Biobank Regensburg, University and University Hospital Regensburg, 93053 Regensburg, Germany; 3 Department of Otorhinolaryngology, Head and Neck Surgery, Medical University of Vienna, 1090 Vienna, Austria; 4 Department of Oral and Maxillofacial Surgery, University Hospital Regensburg, 93053 Regensburg, Germany; 5 Department of Otorhinolaryngology, University Hospital Regensburg, 93053 Regensburg, Germany; 6 Institute of Pathology, University Medical Centre Mannheim, Ruprecht-Karls-University of Heidelberg, 68167 Mannheim, Germany

**Keywords:** head and neck cancer, OSCC, immune microenvironment, pathology, immunohistochemistry, spatial distribution, topology

## Abstract

Background: Evaluating the tumor microenvironment and its influence on clinical management and therapy response is becoming increasingly important. However, only a few studies deal with the spatial distribution of immune cells within the tumor. This study aimed to describe the topology of immune cells in the microenvironment of oral squamous cell carcinoma (OSCC) sectioned by tumor invasion front and tumor center and to test their prognostic relevance regarding patient survival. Methods: A total of 55 OSCC patient specimens were collected retrospectively. The cancer tissue was immunohistochemically stained using an automated tissue stainer Ventana Benchmark Ultra (Roche) and analyzed using discrete expression marker profiles on immune cells. We investigated CD4+ lymphocytes, CD8+ lymphocytes, CD68+ macrophages, CD163+ macrophages, and M1 macrophages regarding their spatial distribution. Results: The statistical analysis revealed that the quantity and distribution of CD4+ (*p* = 0.007), CD8+ (*p* < 0.001), CD68+ (*p* < 0.001), CD163+ cells (*p* = 0.004), and M1 (*p* < 0.001) macrophages were significantly higher at the invasion front compared to the tumor center in all observed cases. However, high and low immune cell counts in the tumor center and invasion front were not associated with overall survival. Conclusion: Our results show two distinct immune microenvironments of the tumor center compared to the invasion front. Future studies are needed to explore how these results can be leveraged to improve patient therapy and outcome.

## 1. Introduction

Oral squamous cell carcinoma (OSCC) is the most common malignancy of the oral cavity [1]. In 2020, the International Agency for Research on Cancer estimated 377,713 new cases and 177,757 deaths worldwide making it the 18th most common cancer [2]. Despite OSCC’s aggressive nature, tremendous progress has been made in prevention, diagnosis, and treatment options in recent decades [3,4]. At present, patients with newly diagnosed OSCC have a 5-year survival of 68%, influenced by various parameters such as anatomical location, tumor stage, pathological grading, and patient age [1,5]. However, therapy options and survival probabilities of advanced OSCC remain poor with a median overall survival (OS) of <1 year [1]. In up to 40% of patients, metastases in the cervical lymph nodes can be detected at an early stage of the disease [3]. Additionally, OSCC has an average risk of 20% for distant metastasis during disease progression [6].

Considering these facts, the investigation of underlying mechanisms to develop treatments is urgently needed. Recent studies have demonstrated an important role of the tumor immune microenvironment (TIME) in metastasis and disease progression [7]. Tumor-associated macrophages (TAMs) represent a major component of the OSCC TIME and have a bipolar role, depending on their activation state [8,9]. M1 macrophages are tumor suppressive and facilitate tumor-specific antigen presentation [10]. In contrast, M2 macrophages (CD163+) are known to reduce radiosensitivity, promote anti-inflammatory response, and play a role in angiogenesis as well as in epithelial-to-mesenchymal transition, a fundamental process of metastasis [10,11]. Multiple studies have shown that different macrophage populations influence carcinogenesis, immune evasion, chemotherapy resistance, metastasis, and disease progression of OSCC [8,10].

The second major immune cell population are tumor-infiltrating lymphocytes (TILs) [12]. CD4+ (regulatory T cells) and CD8+ (effector T cells) T cells are both parts of the adaptive immune system [12]. While CD8+ T cells promote a tumor-suppressive environment, CD4+ T cells have a tumor-promoting effect [13]. Several authors discovered that high CD8+ T-cell densities inside OSCC are associated with increased overall survival in OSCC [12,13,14]. Current evidence suggests that cancer immune cell populations are not randomly distributed but follow a specific pattern [15]. However, more research needs to be carried out to fully understand the complexity of the TIME [15]. To the best of our knowledge, no study has yet investigated the lymphocytes and macrophages in OSCC simultaneously.

The present study aimed to characterize the spatial distribution of immune cells in OSCC. Therefore, we used immunohistochemistry to evaluate the quantity and distribution of CD4+ lymphocytes, CD8+ lymphocytes, CD68+ macrophages, CD163+ macrophages, and M1-macrophages in the tumor center and invasion front in a cohort of 55 OSCC patients. Furthermore, we assessed the prognostic relevance of lymphocytes and macrophages regarding survival according to their spatial distribution.

## 2. Materials and Methods

### 2.1. Patients and Tissue Samples

After achieving a positive ethics vote (12-101-0070), cancer tissue from 55 patients with squamous cell carcinoma of the oral cavity treated at the University Hospital of Regensburg, Germany, were included in this study. The diagnosis of squamous cell carcinoma was reviewed and confirmed. TNM staging was performed according to the UICC guidelines of the 7th edition [16].

### 2.2. Histopathological Analysis

**Figure 1:**
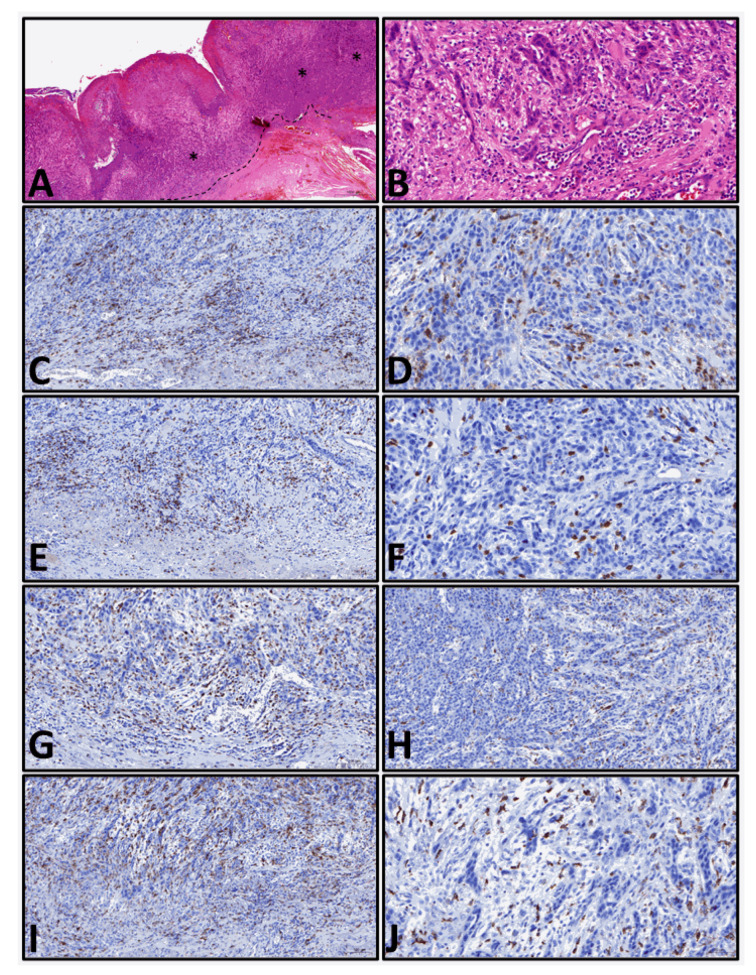
Examples of the evaluated cases. (**A**): Overview of a tissue sample, H.E. 40× magnification (- - - invasion front, * tumor center, H.E.-stain). (**B**): High power field of invasion front, H.E. 400× magnification (H.E.-stain). (**C**,**D**): Immunohistochemical reaction for CD4+ lymphocytes at the invasion front ((**C**), 200× magnification) and tumor center ((**D**), 400× magnification). (**E**,**F**): Immunohistochemical reaction for CD8+ lymphocytes at the invasion front ((**E**), 200× magnification) and tumor center ((**F**), 400× magnification). (**G**,**H**): Immunohistochemical reaction for CD68+ macrophages at the invasion front ((**G**), 200× magnification) and tumor center ((**H**), 200× magnification). (**I**,**J**): Immunohistochemical reaction for CD163+ M2-macrophages at the invasion front ((**I**), 200×) and tumor center ((**J**), 400× magnification). All immunohistochemical reactions mark positive cells in brown.

Tissue from the pathological routine diagnostics was used for histological examination. To characterize the TIME, we chose CD4 (Ventana, anti-CD4 Rabbit Monoclonal Primary Antibody, Clone SP35, dilution: 1:200) and CD8 (Ventana, anti-CD8 Rabbit Monoclonal Primary Antibody, Clone SP57, dilution: 1:200) as marker for lymphocytes, CD68 (Dako Anti-Human CD68, Clone KP1, dilution: 1:200) as a pan marker for macrophages and CD163 (Novocastra, Lyophilized Mouse Monoclonal Antibody CD163, Clone 10D6, dilution: 1:100) as a marker for M2-macrophages. First, a new hematoxylin-eosin slide was prepared, and the tumor was identified (Figure 1A,B). All immunohistochemical stains were performed on tissue sections (4 µm thickness) prepared from formalin-fixed (4% neutral buffered formalin) paraffin-embedded tissue blocks. The procedure is part of the established routine diagnostic at our institute. Briefly, immunohistochemical staining was performed using a Roche Ventana Benchmark Ultra automated slide stainer (Ventana Medical Systems, Roche, France) with the OptiView DAB IHC Detection Kit (Roche, France). The first step of the automatic staining program includes deparaffinization and rehydrating the specimens. Then antigen retrieval was completed by heat treatment lasting for 32 min with Tris-EDTA Borat Puffer (pH 8–8.5). After incubation of the diluted primary antibodies, the nuclei were counterstained with hematoxylin.

All slides were scanned (3DHISTECH Ltd. Pannoramic slide scanner 250) and evaluated using a virtual microscopy software (3DHISTECH Ltd. Case Viewer Ver.2.2). To quantify lymphocytes, ten high power fields (HPF) were identified independently by two pathologists (A.M. & C.B.). The invasion front and tumor center were identified, and the positive cells were counted. The mean values were calculated. The CD68 reaction was used to visualize all macrophages. To evaluate the quantity of the macrophages, ten HPF of the invasion tumor front and intra-tumoral area were examined and the positive cells were counted. The mean value (confidence interval 95%) of macrophages within the tumor front and the intra-tumoral area was calculated. The macrophages were then characterized concerning their subpopulations. M2-macrophages were detected using CD163-antibody. The M2-macrophages were quantified, utilizing the same previously described procedure for quantifying CD68-positive macrophages. The number of M1-macrophages per HPF was calculated through the difference between CD68-positive-macrophages and CD163-positive-M2-macrophages.

### 2.3. Statistical Analysis

For the statistical analysis, all acquired results were documented and statistically evaluated in GraphPad Prism (Version 9.0.0 for MacOS, GraphPad Software, San Diego, CA, USA) and STATA (StataCorp. 2015. Stata Statistical Software: Release 14. College Station, TX, USA: StataCorp LP). We used the Shapiro–Wilk test to test for normal distribution of continuous variables. To investigate the significant differences between the distribution of immune cells in the tumor invasion front and tumor center, a *t*-test or Mann–Whitney-U-test was applied for parametric and nonparametric values, respectively. Spearman’s rank correlation was used to analyze the correlation between immune cell counts in the tumor center and invasion front. Hazard ratios were calculated using the univariable Cox regression models. Overall survival (OS) was defined as the time between primary treatment and death. Survival curves were generated using the Kaplan–Meier estimator. The mean follow-up was 3.9 years (±0.8). All *p*-values ≤ 0.05 were considered statistically significant.

## 3. Results

All cancer tissue samples were able to be analyzed. Patient demographics, histological and clinical characteristics are displayed in Table 1.

Next, we investigated the difference in immune cell numbers between the invasion front and the tumor center. The mean value of CD4+ lymphocytes was 12.04 in the tumor center and 40.79 at the invasion front. The mean value of CD8+ lymphocytes in the tumor center and at the invasion front were 11.72 and 47.93, respectively. The mean values of CD68+ macrophages in the tumor center and invasion front were 14.01 and 44.85, respectively. The mean values of CD163+ M2-macrophages in the tumor center were 7.65 and 30.07 in the invasion front. The mean values of M1-macrophages in the tumor center and invasion front were 6.35 and 14.77, respectively (Table 2).

A *t*-test for paired parameters and Spearman’s rank correlation comparing mean values and correlation between the tumor invasion front and tumor center showed the following results: All examined parameters showed significantly more immune cells in the invasion front than the tumor center. In detail, the CD4+ lymphocytes, CD8+ lymphocytes, CD68+ macrophages, CD163+ M2-macrophages, and M1-macrophages were all significantly elevated in the invasion front (*p* < 0.001/*p* < 0.001/*p* < 0.001/*p* < 0.001/*p* < 0.001) (Table 3). The correlation factor between invasion front and tumor center for CD4+ lymphocytes, CD8+ lymphocytes, CD68+ macrophages, CD163+ M2-macrophages and M1-macrophages were 0.3841, 0.6587, 0.5831, and −0.0658 respectively.

Finally, we evaluated the prognostic relevance of immune cell counts on OS. Therefore, we dichotomized immune cell counts along the mean value of each cell type. All patient characteristics of dichotomized immune cell groups are shown in Supplementary Tables S1–S5. The univariate Cox regression of patient demographics and histological characteristics are shown in Table 4.

**Figure 2:**
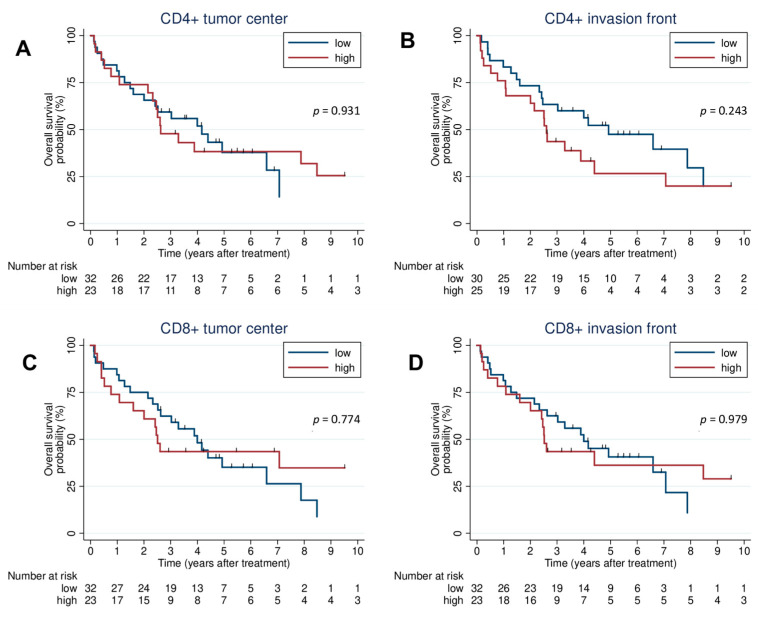
Survival analysis. Kaplan–Meier plots of OS for CD4+ tumor center (**A**), CD4+ invasion front (**B**), CD8+ tumor center (**C**), CD8+ invasion front (**D**). OS: overall survival.

There was no significant difference in OS between high CD4+ and low CD4+ cell in the tumor center (HR: 0.97; 95% CI: 0.49–1.91; *p* = 0.931) and the invasion front (HR: 1.48; 95% CI: 0.77–2.86; *p* = 0.242). Moreover, there was no difference in OS between high and low CD8+ cell counts in the tumor center (HR: 0.91; 95% CI: 0.46–1.79; *p* = 0.774) and the invasion front (HR: 1.01; 95% CI: 0.51–1.99; *p* = 0.979). The Kaplan–Meier curves for lymphocytes are shown in Figure 2.

**Figure 3:**
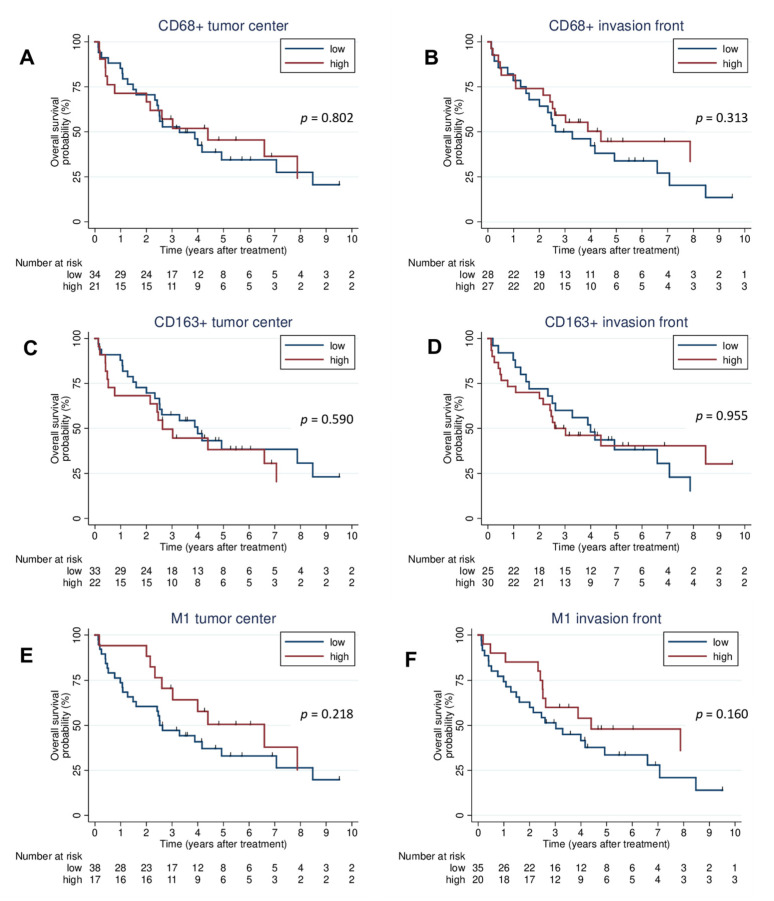
Survival analysis. Kaplan–Meier plots of OS for CD68+ in the tumor center (**A**) and the invasion front (**B**), CD163+ in the tumor center (**C**) and the invasion front (**D**), and M1 macrophages in tumor center (**E**) and invasion front (**F**). OS: overall survival.

High and low cell counts of CD68+ cells in the tumor center (HR: 0.92; 95% CI: 0.46–1.81; *p* = 0.803), CD68+ cells in the invasion front (HR: 0.71; 95% CI: 0.37–1.38; *p* = 0.317), CD163+ cells in the tumor center (HR: 1.20; 95% CI: 0.62–2.34; *p* = 0.588), and CD163+ cells in the invasion front (HR: 0.98; 95% CI: 0.51–1.89; *p* = 0.955) showed no prognostic relevance in OS. Furthermore, regarding M1 macrophages, there was no difference in OS between high and low groups in the tumor center (HR: 0.64; 95% CI: 0.31–1.33; *p* = 0.231) and the invasion front (HR: 0.60; 95% CI: 0.29–1.22; *p* = 0.160). The Kaplan–Meier curves for macrophages are shown in Figure 3.

## 4. Discussion

The present study characterized the TIME regarding the spatial distribution of lymphocytes and macrophages. All immune cells were significantly elevated in the tumor margin when compared to the tumor center. However, none of the immune cells were prognostic for OS. Specific patterns of immune cell distributions have been observed in various human cancer types [17,18]. However, the former studies focused on quantification of different types of lymphocytes or the different polarization mechanisms of macrophages. In the present study, we analyzed both lymphocytes and macrophages and the effect of their interplay. Thus, we define the presence of macrophages and the different types of lymphocytes together with the morphological background as the TIME.

CD4+ and CD8+ cells are the dominant TILs of the OSCC microenvironment [19]. In our study cohort, we observed significantly lower levels of CD4+ and CD8+ cells in the tumor center compared to the invasion front. This represents a highly active immune environment and is indicative of an acute immunological response [20]. To date, it is not clear if this phenomenon is an immunological response against the tumor tissue or a protective mechanism of the tumor itself. We did not observe any association of CD4+ and CD8+ cell numbers in the tumor center or in the invasion front with OS. However, many solid tumors, including colorectal cancer, breast cancer, and non-small cell lung cancer, have shown a significant correlation between TIL quantities and patient outcomes [21,22]. This correlation has also been observed in head and neck squamous cell carcinoma (HNSCC), although the results have been inconsistent depending on the anatomical location [23,24]. Accordingly, the clinical implementation of TIL analysis in the routine practice of pathologists is far from reached [25]. Only a few studies have investigated TILs in OSCC while paying attention to the cellular topology. Watanabe et al. first examined TILs in 87 OSCC patients based on their spatial distribution. Low numbers of CD8+ cells in the tumor center and the invasion margin were associated with worse survival, while this association only remained significant in early stage OSCC [26]. Zhou et al. showed that CD3+, a pan lymphocyte marker, and CD8+ lymphocyte quantities differed inside the tumor center and the invasion margin in OSCC. In line with our results, the authors observed more CD3+ and CD8+ cells in the tumor center compared to that in the invasion margin in all samples. Furthermore, the authors investigated whether CD3+ and CD8+ topology could be used as prognostic survival marker. Interestingly, only high CD8+ cells in the tumor center were associated with an improved survival, but not in the tumor margins [13]. This may be linked to the fact that tumors with strong immunogenic core are more responsive to therapy and tend to have a better outcome [27].

In our study cohort, we observed statistically significantly more CD163+ M2-macrophages in the tumor margins than in the tumor center. The activation of macrophages is one of the first reactions of the immune system to pathogens [20]. Our results revealed a high number of macrophages which invade the tumor margin. Especially in correlation with high lymphocyte count, this strengthens the theory of an overall highly immunogenic microenvironment in the tumor margin. Interestingly, we observed almost complete polarization of macrophages in CD163+ M2-macrophages in the tumor margin. It is well established that the polarization on macrophages can significantly impact tumor behavior [28]. However, we did not find a correlation of CD68+, CD163+, and M1-macrophages with OS. The literature on the topology of macrophages in OSCC is limited. In 2021, Hori et al. studied CD163+ macrophages in the tumor center and invasion margin. While they assessed the spatial distribution resulting in higher CD163+ cell density in the invasion front, it was not the focus of their investigation so ratios between tumor center and invasion were not tested for significance. The distribution and prognostic relevance of TAMs seem to differ greatly according to the tumor entity [29]. Huang et al. studied the spatial heterogeneity of CD68+ and CD163+ M2-macrophage populations in gastric cancer tissue using multiplex staining. They consistently observed more M2-macrophages in the tumor center than in the tumor margins, whereas we did not observe this correlation in our study population. Since CD163+ M2-macrophages are involved in the clearance of dead cells and tissue remodeling, the authors suggest that high numbers may reflect an enhanced immunological response in these tumors [18]. HNSCC, and particularly OSCC, are known to have a strong immune-suppressive environment [30], which could explain the low levels of CD163+ cells in the tumor center observed in our cohort. Furthermore, a recent study by Zheng et al. evaluated the spatial distribution and density of M1- and CD163+ M2-macrophages in non-small cell lung cancer using multiplex immunofluorescence staining and RNA-seq. The proximity to tumor cells alters gene expression and is a negative prognosticator for survival. Moreover, they observed a predominance of M2-macrophages over M1 in the invasion margin, which is in line with our results. The clinical importance of these findings is highlighted by the positive prognostic correlation of CD163+ M2-macrophages in the invasion front on patient outcomes [31]. In the present study, however, high M1-macrophages in the invasion front showed a slight trend toward improved OS. There is evidence that M1-macrophages express a tumor-suppressing phenotype [32]. Despite this slight trend, the results are not significant and larger studies with greater statistical power are warranted to draw more robust conclusion. Regarding data of OSCC, in 2015, Wolf et al. showed that higher CD68+ macrophage quantity is a negative prognosticator in OSCC [33]. In contrast, a recent meta-analysis by Hadler-Olsen et al. including 33 studies on all types of immune cells as prognostic markers in OSCC, evaluated CD163+ M2 macrophages as the most promising predictor for survival in OSCC patients. The authors noted methodological deficiencies in most included studies, making reliable conclusions difficult [34]. Another meta-analysis demonstrated the prognostic value of stromal CD163+ but not CD68+ [32]. Both studies tried to account the difference between stromal and intratumor macrophage population. However, most included studies only assessed one subsite of tumor tissue which aggravates comparability.

It is well-established that lymphocytes and macrophages influence tumor progression based on their quantity, phenotype, and the type of cancer they are associated with. Although macrophages and lymphocytes have been the target of intensive research, little attention has been paid to the interactions between both cell populations in cancer tissue. However, it is widely known that its bidirectional interactions are essential for differentiation, up- and downregulation and immune response and cancer development [20]. In this context, immune cell topology arises as an important dimension for gaining insights into tumor biology and disease progression and may ultimately influence new treatment options [35].

Considering the differences in immune cell distribution between different cancer types and the inconsistent results of microenvironment characterization in OSCC tumor tissue, it is crucial to ensure the comparability of future studies. One way to achieve this goal is the use of large, standardized biobanks, which can provide high-quality, homogeneous tissue samples across different tumor entities. Since it is important to relate morphological criteria to clinical data in future studies, structured tumor biobanks should be established by standard as already described in traumatology [36].

Surgical resection is the primary treatment option for early and late-stage OSCC. If OSCC is detected in stages III and IV, surgery is typically combined with chemoradiation or adjuvant radiation [3]. The response to chemo- and radiotherapy is closely linked to an efficient immune response [37]. Herter et al. analyzed the immune cell population of 34 patients with cervical cancer after chemoradiation and found significantly decreased CD4+ and CD8+ cells and increased levels of macrophages inside the radiated cancer tissue. However, they did not consider immune cell topology [38]. In contrast, Gartrell et al. detected increased levels of CD4+ and CD8+ cells and decreased levels of CD68+ macrophages in chemo-radiated patients with pancreatic ductal adenocarcinoma [39]. Recent pathohistological studies have shown that recurrent HNSCC tumors treated with chemoradiotherapy have overall compromised immune cell populations, which may contribute to poor therapy response and prognosis [40]. We also observed this immunocompromised microenvironment in our cohort. It is essential to mention that, to our knowledge, no study to date has made correlations between chemoradiotherapy and the spatial distribution of immune cells in OSCC. In the field of immunotherapy, understanding the TIME becomes even more critical. In 2017, nivolumab, the first antibody drug targeting the PD-1/PD-L1 immune checkpoint axis, received approval from the European Commission for treatment of platin-refractory recurrent HNSCC. Soon after, in 2019, the FDA approved pembrolizumab for patients with metastatic or unresectable, recurrent HNSCC [41,42,43]. Treatment efficacy of these drugs relies on the PD-L1 expression of tumor cells. The current estimated one-year survival rate is 36%, and resistance mechanisms are still unknown [44]. Blatt et al. analyzed the different PD-L1 expressions between oral and oropharyngeal SCC [45]. Additionally, a study by Hirshoren et al. revealed that PD-L1 expression is unevenly distributed within the tumor itself [46]. This underlines the clinical relevance of our findings since biopsies of different tumor regions can lead to different pathological evaluation.

There are several limitations to our study. In recent years, the paradigm of M1 and M2 dichotomy in macrophages has been called into question, with increasing evidence that we are dealing with a continuum of surface marker expression rather than two distinct phenotypes [41,42]. Furthermore, although CD68 is recognized as a pan-macrophage marker, low expressions of CD68 can also be found in other cell types such as fibroblasts, endothelial cells, and tumor cells [16,43]. However, we consider this limitation not as significant since evaluation was performed by two consultant pathologists. In addition to spatial distribution, spatial density is important, which we did not assess [26].

## 5. Conclusions

The present study shows that the spatial distribution of CD4+, CD8+, CD68+, and CD163+ immune cells differs between the tumor center and invasion front of OSCC. While the invasion front shows high immune cell counts, the tumor center is sparse in immune cells. Especially in light of emerging treatment modalities like immunotherapy, our results underline the importance of a holistic observation of the tumor tissue. Taking biopsies for routine clinical workup or research must be done with consideration of the TIME since it influences clinical decision-making and research results. The region inside the tumor tissue, where biopsies are taken from, is critical and must be stated carefully. Further prospective studies and genetic expression data are warranted to validate our results and explore how these results can be leveraged to improve patient therapy and outcome.

## Figures and Tables

**Table 1:** Patient characteristics of the OSCC cohort.

	Total	
Variables/Categories	n	(%)
Number of patients	55	(100.0%)
Age		
≥70	20	(36.4%)
<70	35	(63.6%)
Gender		
male	39	(70.9%)
female	16	(29.1%)
Tumor spread (T-classification, UICC 7th edition)		
T1	20	(36.4%)
T2	15	(27.3%)
T3	7	(12.7%)
T4	13	(23.6%)
Nodal metastasis		
no	30	(54.5%)
yes	25	(45.5%)
Metastasis		
unknown	8	(14.5%)
no spread	40	(72.7%)
any spread	7	(12.7%)
Tumor grade		
1	2	(3.6%)
2	44	(80.0%)
3	9	(16.4%)
Stage		
I	15	(27.3%)
II	6	(10.9%)
III	11	(20.0%)
IV	23	(41.8%)

**Table 2:** Mean, maximum, minimum, and standard deviation of evaluated surface marker are shown. Min., minimum; Max., maximum; SD, standard deviation.

	Min	Max	Mean	SD
CD4+ lymphocytes tumor center	2.2	33.4	12.04	7.68
CD4+ lymphocytes invasion front	4.0	100.2	40.79	18.74
CD8+ lymphocytes tumor center	0.1	45.1	11,72	10.79
CD8+ lymphocytes invasion front	1.4	106.8	47.93	23.62
CD68+ macrophages tumor center	0.6	46.1	14.01	10.20
CD68+ macrophages invasion front	13.5	75.2	44.85	15.76
CD163+ M2-macrophages tumor center	0	23.8	7.65	6.72
CD163+ M2-macrophages invasion front	0	55.7	30.07	13.69
M1-macrophages tumor center	0	28.6	6.35	7.14
M1-macrophages invasion front	0.1	52.4	14.77	11.00

**Table 3:** Paired *t*-test and Spearman’s correlation of immune cell counts for tumor center vs. invasion front.

		*t*-Test	Correlation	
	n	*p*-Value	Rho	*p*-Value
CD4+ lymphocytes tumor center and CD4+ lymphocytes invasion front	55	<0.001	0.384	0.004
CD8+ lymphocytes tumor center and CD8+ lymphocytes invasion front	55	<0.001	0.659	<0.001
CD68+ macrophages tumor center and CD68+ macrophages invasion front	55	<0.001	0.502	<0.001
CD163+ M2-macrophages tumor center and CD163+ M2-macrophages invasion front	55	<0.001	0.583	<0.001
M1-macrophages tumor center and M1-macrophages invasion front	55	<0.001	−0.066	0.633

**Table 4:** Univariate Cox regression of all patient characteristics.

Variables/Levels		OS		
	n	HR	(95% CI)	*p*-Value
CD4+ tumor center	55			
high vs. low (ref)		0.97	(0.49–1.91)	0.931
CD4+ invasion front	55			
high vs. low (ref)		1.48	(0.77–2.86)	0.242
CD8+ tumor center	55			
high vs. low (ref)		0.91	(0.46–1.79)	0.774
CD8+ invasion front	55			
high vs. low (ref)		1.01	(0.51–1.99)	0.979
CD68+ tumor center	55			
high vs. low (ref)		0.92	(0.46–1.81)	0.803
CD68+ invasion front	55			
high vs. low (ref)		0.71	(0.37–1.38)	0.317
CD163+ tumor center	55			
high vs. low (ref)		1.20	(0.62–2.34)	0.588
CD163+ invasion front	55			
high vs. low (ref)		0.98	(0.51–1.89)	0.955
M1 tumor center	55			
high vs. low (ref)		0.64	(0.31–1.33)	0.231
M1 invasion front	55			
high vs. low (ref)		0.60	(0.29–1.22)	0.160

## Data Availability

Not applicable.

## Supplementary Materials

The following supporting information can be downloaded at: https://www.mdpi.com/article/10.3390/jcm12041704/s1, Table S1: Patient characteristics according to dichotomized CD4+ tumor center and CD4+ invasion front cell counts; Table S2: Patient characteristics according to dichotomized CD8+ tumor center and CD8+ invasion front cell counts; Table S3: Patient characteristics according to dichotomized CD68+ tumor center and CD68+ invasion front cell counts; Table S4: Patient characteristics according to dichotomized CD163+ tumor center and CD163+ invasion front cell counts; Table S5: Patient characteristics according to dichotomized M1 tumor center and M1 invasion front cell counts.

## Abbreviations

HNSCC: head and neck squamous cell carcinoma; HPF: high power field; OS: overall survival, OSCC: oral squamous cell carcinoma; TAM: tumor associated macrophage; TIL: tumor infiltrating lymphocytes; TIME: tumor immune microenvironment.

## References

[1] Chamoli A., Gosavi A.S., Shirwadkar U.P., Wangdale K.V., Behera S.K., Kurrey N.K., Kalia K., Mandoli A. (2021). Overview of Oral Cavity Squamous Cell Carcinoma: Risk Factors, Mechanisms, and Diagnostics. Oral Oncol..

[2] Sung H., Ferlay J., Siegel R.L., Laversanne M., Soerjomataram I., Jemal A., Bray F. (2021). Global Cancer Statistics 2020: GLOBOCAN Estimates of Incidence and Mortality Worldwide for 36 Cancers in 185 Countries. CA Cancer J. Clin..

[3] Chi A.C., Day T.A., Neville B.W. (2015). Oral Cavity and Oropharyngeal Squamous Cell Carcinoma--an Update. CA Cancer J. Clin..

[4] Amit M., Yen T.-C., Liao C.-T., Chaturvedi P., Agarwal J.P., Kowalski L.P., Ebrahimi A., Clark J.R., Kreppel M., Zöller J. (2013). Improvement in Survival of Patients with Oral Cavity Squamous Cell Carcinoma: An International Collaborative Study. Cancer.

[5] Cancer of the Oral Cavity and Pharynx—Cancer Stat Facts. https://seer.cancer.gov/statfacts/html/oralcav.html.

[6] Bauml J.M., Aggarwal C., Cohen R.B. (2019). Immunotherapy for Head and Neck Cancer: Where Are We Now and Where Are We Going?. Ann. Transl. Med..

[7] Chew V., Toh H.C., Abastado J.-P. (2012). Immune Microenvironment in Tumor Progression: Characteristics and Challenges for Therapy. J. Oncol..

[8] Kalogirou E.M., Tosios K.I., Christopoulos P.F. (2021). The Role of Macrophages in Oral Squamous Cell Carcinoma. Front. Oncol..

[9] Murray P.J., Allen J.E., Biswas S.K., Fisher E.A., Gilroy D.W., Goerdt S., Gordon S., Hamilton J.A., Ivashkiv L.B., Lawrence T. (2014). Macrophage Activation and Polarization: Nomenclature and Experimental Guidelines. Immunity.

[10] Shigeoka M., Koma Y.-I., Nishio M., Akashi M., Yokozaki H. (2021). Alteration of Macrophage Infiltrating Compartment: A Novel View on Oral Carcinogenesis. Pathobiology.

[11] Corliss B.A., Azimi M.S., Munson J.M., Peirce S.M., Murfee W.L. (2016). Macrophages: An Inflammatory Link Between Angiogenesis and Lymphangiogenesis. Microcirculation.

[12] Nguyen N., Bellile E., Thomas D., McHugh J., Rozek L., Virani S., Peterson L., Carey T.E., Walline H., Moyer J. (2016). Tumor Infiltrating Lymphocytes and Survival in Patients with Head and Neck Squamous Cell Carcinoma. Head Neck.

[13] Zhou C., Wu Y., Jiang L., Li Z., Diao P., Wang D., Zhang W., Liu L., Wang Y., Jiang H. (2018). Density and Location of CD3+ and CD8+ Tumor-Infiltrating Lymphocytes Correlate with Prognosis of Oral Squamous Cell Carcinoma. J. Oral Pathol. Med..

[14] Li F., Li C., Cai X., Xie Z., Zhou L., Cheng B., Zhong R., Xiong S., Li J., Chen Z. (2021). The Association between CD8+ Tumor-Infiltrating Lymphocytes and the Clinical Outcome of Cancer Immunotherapy: A Systematic Review and Meta-Analysis. EClinicalMedicine.

[15] Baghban R., Roshangar L., Jahanban-Esfahlan R., Seidi K., Ebrahimi-Kalan A., Jaymand M., Kolahian S., Javaheri T., Zare P. (2020). Tumor Microenvironment Complexity and Therapeutic Implications at a Glance. Cell Commun. Signal..

[16] Sobin L.H., Gospodarowicz M.K., Wittekind C. (2011). TNM Classification of Malignant Tumours.

[17] Väyrynen J.P., Haruki K., Väyrynen S.A., Lau M.C., Dias Costa A., Borowsky J., Zhao M., Ugai T., Kishikawa J., Akimoto N. (2021). Prognostic Significance of Myeloid Immune Cells and Their Spatial Distribution in the Colorectal Cancer Microenvironment. J. Immunother. Cancer.

[18] Huang Y.-K., Wang M., Sun Y., Di Costanzo N., Mitchell C., Achuthan A., Hamilton J.A., Busuttil R.A., Boussioutas A. (2019). Macrophage Spatial Heterogeneity in Gastric Cancer Defined by Multiplex Immunohistochemistry. Nat. Commun..

[19] Almangush A., Leivo I., Mäkitie A.A. (2020). Overall Assessment of Tumor-Infiltrating Lymphocytes in Head and Neck Squamous Cell Carcinoma: Time to Take Notice. Acta Otolaryngol..

[20] Biswas S.K., Mantovani A. (2010). Macrophage Plasticity and Interaction with Lymphocyte Subsets: Cancer as a Paradigm. Nat. Immunol..

[21] Paijens S.T., Vledder A., de Bruyn M., Nijman H.W. (2021). Tumor-Infiltrating Lymphocytes in the Immunotherapy Era. Cell. Mol. Immunol..

[22] Hendry S., Salgado R., Gevaert T., Russell P.A., John T., Thapa B., Christie M., van de Vijver K., Estrada M.V., Gonzalez-Ericsson P.I. (2017). Assessing Tumor-Infiltrating Lymphocytes in Solid Tumors: A Practical Review for Pathologists and Proposal for a Standardized Method from the International Immuno-Oncology Biomarkers Working Group: Part 2: TILs in Melanoma, Gastrointestinal Tract Carcinomas, Non-Small Cell Lung Carcinoma and Mesothelioma, Endometrial and Ovarian Carcinomas, Squamous Cell Carcinoma of the Head and Neck, Genitourinary Carcinomas, and Primary Brain Tumors. Adv. Anat. Pathol..

[23] Chatzopoulos K., Kotoula V., Manoussou K., Markou K., Vlachtsis K., Angouridakis N., Nikolaou A., Vassilakopoulou M., Psyrri A., Fountzilas G. (2020). Tumor Infiltrating Lymphocytes and CD8+ T Cell Subsets as Prognostic Markers in Patients with Surgically Treated Laryngeal Squamous Cell Carcinoma. Head Neck Pathol..

[24] Prasetyo A., Budiman J., Sadhana U. (2021). The Relationship between Tumor-Infiltrating Lymphocytes (TILs) and Nasopharyngeal Carcinoma (NPC): A Systematic Review. Iran J. Otorhinolaryngol..

[25] De Meulenaere A., Vermassen T., Aspeslagh S., Vandecasteele K., Rottey S., Ferdinande L. (2017). TILs in Head and Neck Cancer: Ready for Clinical Implementation and Why (Not)?. Head Neck Pathol..

[26] Watanabe Y., Katou F., Ohtani H., Nakayama T., Yoshie O., Hashimoto K. (2010). Tumor-Infiltrating Lymphocytes, Particularly the Balance between CD8(+) T Cells and CCR4(+) Regulatory T Cells, Affect the Survival of Patients with Oral Squamous Cell Carcinoma. Oral Surg. Oral Med. Oral Pathol. Oral Radiol. Endod..

[27] Anitei M.-G., Zeitoun G., Mlecnik B., Marliot F., Haicheur N., Todosi A.-M., Kirilovsky A., Lagorce C., Bindea G., Ferariu D. (2014). Prognostic and Predictive Values of the Immunoscore in Patients with Rectal Cancer. Clin. Cancer Res..

[28] Rhee I. (2016). Diverse Macrophages Polarization in Tumor Microenvironment. Arch. Pharm. Res..

[29] Hori Y., Kubota A., Yokose T., Furukawa M., Matsushita T., Katsumata N., Oridate N. (2021). Prognostic Role of Tumor-Infiltrating Lymphocytes and Tumor Budding in Early Oral Tongue Carcinoma. Laryngoscope.

[30] Spenlé C., Loustau T., Murdamoothoo D., Erne W., Beghelli-de la Forest Divonne S., Veber R., Petti L., Bourdely P., Mörgelin M., Brauchle E.-M. (2020). Tenascin-C Orchestrates an Immune-Suppressive Tumor Microenvironment in Oral Squamous Cell Carcinoma. Cancer Immunol. Res..

[31] Zheng X., Weigert A., Reu S., Guenther S., Mansouri S., Bassaly B., Gattenlöhner S., Grimminger F., Pullamsetti S., Seeger W. (2020). Spatial Density and Distribution of Tumor-Associated Macrophages Predict Survival in Non-Small Cell Lung Carcinoma. Cancer Res..

[32] Troiano G., Caponio V.C.A., Adipietro I., Tepedino M., Santoro R., Laino L., Lo Russo L., Cirillo N., Lo Muzio L. (2019). Prognostic Significance of CD68+ and CD163+ Tumor Associated Macrophages in Head and Neck Squamous Cell Carcinoma: A Systematic Review and Meta-Analysis. Oral Oncol..

[33] Wolf G.T., Chepeha D.B., Bellile E., Nguyen A., Thomas D., McHugh J. (2015). University of Michigan Head and Neck SPORE Program Tumor Infiltrating Lymphocytes (TIL) and Prognosis in Oral Cavity Squamous Carcinoma: A Preliminary Study. Oral Oncol..

[34] Hadler-Olsen E., Wirsing A.M. (2019). Tissue-Infiltrating Immune Cells as Prognostic Markers in Oral Squamous Cell Carcinoma: A Systematic Review and Meta-Analysis. Br. J. Cancer.

[35] Fridman W.H., Pagès F., Sautès-Fridman C., Galon J. (2012). The Immune Contexture in Human Tumours: Impact on Clinical Outcome. Nat. Rev. Cancer.

[36] Brochhausen M., Whorton J.M., Zayas C.E., Kimbrell M.P., Bost S.J., Singh N., Brochhausen C., Sexton K.W., Blobel B. (2022). Assessing the Need for Semantic Data Integration for Surgical Biobanks-A Knowledge Representation Perspective. J. Pers. Med..

[37] Merlano M.C., Denaro N., Galizia D., Ruatta F., Occelli M., Minei S., Abbona A., Paccagnella M., Ghidini M., Garrone O. (2022). How Chemotherapy Affects the Tumor Immune Microenvironment: A Narrative Review. Biomedicines.

[38] Herter J.M., Kiljan M., Kunze S., Reinscheid M., Ibruli O., Cai J., Niu L., Heßelmann I., Trommer M., Herter-Sprie G.S. (2022). Influence of Chemoradiation on the Immune Microenvironment of Cervical Cancer Patients. Strahlenther. Onkol..

[39] Gartrell R.D., Enzler T., Kim P.S., Fullerton B.T., Fazlollahi L., Chen A.X., Minns H.E., Perni S., Weisberg S.P., Rizk E.M. (2022). Neoadjuvant Chemoradiation Alters the Immune Microenvironment in Pancreatic Ductal Adenocarcinoma. Oncoimmunology.

[40] Watermann C., Pasternack H., Idel C., Ribbat-Idel J., Brägelmann J., Kuppler P., Offermann A., Jonigk D., Kühnel M.P., Schröck A. (2021). Recurrent HNSCC Harbor an Immunosuppressive Tumor Immune Microenvironment Suggesting Successful Tumor Immune Evasion. Clin. Cancer Res..

[41] Burtness B., Harrington K.J., Greil R., Soulières D., Tahara M., de Castro G., Psyrri A., Basté N., Neupane P., Bratland Å. (2019). Pembrolizumab Alone or with Chemotherapy versus Cetuximab with Chemotherapy for Recurrent or Metastatic Squamous Cell Carcinoma of the Head and Neck (KEYNOTE-048): A Randomised, Open-Label, Phase 3 Study. Lancet.

[42] Ferris R.L., Blumenschein G., Fayette J., Guigay J., Colevas A.D., Licitra L., Harrington K., Kasper S., Vokes E.E., Even C. (2016). Nivolumab for Recurrent Squamous-Cell Carcinoma of the Head and Neck. N. Engl. J. Med..

[43] Cohen E.E.W., Bell R.B., Bifulco C.B., Burtness B., Gillison M.L., Harrington K.J., Le Q.-T., Lee N.Y., Leidner R., Lewis R.L. (2019). The Society for Immunotherapy of Cancer Consensus Statement on Immunotherapy for the Treatment of Squamous Cell Carcinoma of the Head and Neck (HNSCC). J. Immunother. Cancer.

[44] Tormoen G.W., Crittenden M.R., Gough M.J. (2018). Role of the Immunosuppressive Microenvironment in Immunotherapy. Adv. Radiat. Oncol..

[45] Blatt S., Krüger M., Rump C., Zimmer S., Sagheb K., Künzel J. (2022). Differences in PD-L1 Expression between Oral and Oropharyngeal Squamous Cell Carcinoma. PLoS ONE.

[46] Hirshoren N., Al-Kharouf I., Weinberger J.M., Eliashar R., Popovtzer A., Knaanie A., Fellig Y., Neuman T., Meir K., Maly A. (2021). Spatial Intratumoral Heterogeneity Expression of PD-L1 Antigen in Head and Neck Squamous Cell Carcinoma. Oncology.

