# Supplementary material for: Tumor Immune Microenvironment Heterogeneity at the Invasion Front and Tumor Center in Oral Squamous Cell Carcinoma as a Perspective of Managing This Cancer Entity
Source: J Clin Med. 2023 Feb 20;12(4):1704. doi: 10.3390/jcm12041704 (PMC9958892; doi:10.3390/jcm12041704)
Supplement: Supplementary file 1 [file jcm-12-01704-s001.zip › jcm-2132419-supplementary.pdf]

Table S1. Patient characteristics according to dichotomized CD4+ tumor center and CD4+ invasion front cell counts.

	CD4+ tumor center				CD4+ invasion front			
	low		high		low		high	
Variables/Categories	n	(%)	n	(%)	n	(%)	n	(%)
**Number of patients**	32	(100.0%)	23	(100.0%)	30	(100.0%)	25	(100.0%)
**Age**								
≥70	13	(40.6%)	7	(30.4%)	10	(33.3%)	10	(40.0%)
<70	19	(59.4%)	16	(69.6%)	20	(66.7%)	15	(60.0%)
**Gender**								
male	20	(62.5%)	19	(82.6%)	20	(66.7%)	19	(76.0%)
female	12	(37.5%)	4	(17.4%)	10	(33.3%)	6	(24.0%)
**Tumor spread**								
1	10	(31.3%)	10	(43.5%)	11	(36.7%)	9	(36.0%)
2	7	(21.9%)	8	(34.8%)	7	(23.3%)	8	(32.0%)
3	4	(12.5%)	3	(13.0%)	3	(10.0%)	4	(16.0%)
4	11	(34.4%)	2	(8.7%)	9	(30.0%)	4	(16.0%)
**Nodal metastasis**								
0	19	(59.4%)	11	(47.8%)	19	(63.3%)	11	(44.0%)
+	13	(40.6%)	12	(52.2%)	11	(36.7%)	14	(56.0%)
**Metastasis**								
no spread	25	(78.1%)	15	(65.2%)	23	(76.7%)	17	(68.0%)
any spread	2	(6.3%)	5	(21.7%)	2	(6.7%)	5	(20.0%)
unknown	5	(15.6%)	3	(13.0%)	5	(16.7%)	3	(12.0%)
**Grade**								
1	2	(6.3%)	0	(0.0%)	1	(3.3%)	1	(4.0%)
2	25	(78.1%)	19	(82.6%)	24	(80.0%)	20	(80.0%)
3	5	(15.6%)	4	(17.4%)	5	(16.7%)	4	(16.0%)
**Stage**								
I	7	(21.9%)	8	(34.8%)	10	(33.3%)	5	(20.0%)
II	4	(12.5%)	2	(8.7%)	3	(10.0%)	3	(12.0%)
III	7	(21.9%)	4	(17.4%)	6	(20.0%)	5	(20.0%)
IV	14	(43.8%)	9	(39.1%)	11	(36.7%)	12	(48.0%)

Table S2. Patient characteristics according to dichotomized CD8+ tumor center and CD8+ invasion front cell counts.

	CD8+ tumor center				CD8+ invasion front			
	low		high		low		high	
Variables/Categories	n	(%)	n	(%)	n	(%)	n	(%)
**Number of patients**	32	(100.0%)	23	(100.0%)	32	(100.0%)	23	(100.0%)
**Age**								
≥70	16	(50.0%)	4	(17.4%)	14	(43.8%)	6	(26.1%)
<70	16	(50.0%)	19	(82.6%)	18	(56.3%)	17	(73.9%)
**Gender**								
male	24	(75.0%)	15	(65.2%)	20	(62.5%)	19	(82.6%)
female	8	(25.0%)	8	(34.8%)	12	(37.5%)	4	(17.4%)
**Tumor spread**								
1	11	(34.4%)	9	(39.1%)	13	(40.6%)	7	(30.4%)
2	8	(25.0%)	7	(30.4%)	8	(25.0%)	7	(30.4%)
3	3	(9.4%)	4	(17.4%)	4	(12.5%)	3	(13.0%)
4	10	(31.3%)	3	(13.0%)	7	(21.9%)	6	(26.1%)
**Nodal metastasis**								
0	16	(50.0%)	14	(60.9%)	16	(50.0%)	14	(60.9%)
+	16	(50.0%)	9	(39.1%)	16	(50.0%)	9	(39.1%)
**Metastais**								
no spread	21	(65.6%)	19	(82.6%)	24	(75.0%)	16	(69.6%)
any spread	6	(18.8%)	1	(4.3%)	4	(12.5%)	3	(13.0%)
unknown	5	(15.6%)	3	(13.0%)	4	(12.5%)	4	(17.4%)
**Grade**								
1	1	(3.1%)	1	(4.3%)	1	(3.1%)	1	(4.3%)
2	24	(75.0%)	20	(87.0%)	27	(84.4%)	17	(73.9%)
3	7	(21.9%)	2	(8.7%)	4	(12.5%)	5	(21.7%)
**Stage**								
I	7	(21.9%)	8	(34.8%)	8	(25.0%)	7	(30.4%)
II	2	(6.3%)	4	(17.4%)	2	(6.3%)	4	(17.4%)
III	6	(18.8%)	5	(21.7%)	9	(28.1%)	2	(8.7%)
IV	17	(53.1%)	6	(26.1%)	13	(40.6%)	10	(43.5%)

Table S3. Patient characteristics according to dichotomized CD68+ tumor center and CD68+ invasion front cell counts.

	CD68+ tumor center				CD68+ invasion front			
	low		high		low		high	
Variables/Categories	n	(%)	n	(%)	n	(%)	n	(%)
**Number of patients**	34	(100.0%)	21	(100.0%)	28	(100.0%)	27	(100.0%)
**Age**								
≥70	11	(32.4%)	9	(42.9%)	11	(39.3%)	9	(33.3%)
<70	23	(67.6%)	12	(57.1%)	17	(60.7%)	18	(66.7%)
**Gender**								
male	25	(73.5%)	14	(66.7%)	19	(67.9%)	20	(74.1%)
female	9	(26.5%)	7	(33.3%)	9	(32.1%)	7	(25.9%)
**Tumor spread**								
1	11	(32.4%)	9	(42.9%)	11	(39.3%)	9	(33.3%)
2	9	(26.5%)	6	(28.6%)	7	(25.0%)	8	(29.6%)
3	3	(8.8%)	4	(19.0%)	3	(10.7%)	4	(14.8%)
4	11	(32.4%)	2	(9.5%)	7	(25.0%)	6	(22.2%)
**Nodal metastasis**								
0	16	(47.1%)	14	(66.7%)	14	(50.0%)	16	(59.3%)
+	18	(52.9%)	7	(33.3%)	14	(50.0%)	11	(40.7%)
**Metastasis**								
no spread	26	(76.5%)	14	(66.7%)	22	(78.6%)	18	(66.7%)
any spread	5	(14.7%)	2	(9.5%)	4	(14.3%)	3	(11.1%)
unknown	3	(8.8%)	5	(23.8%)	2	(7.1%)	6	(22.2%)
**Grade**								
1	0	(0.0%)	2	(9.5%)	0	(0.0%)	2	(7.4%)
2	27	(79.4%)	17	(81.0%)	22	(78.6%)	22	(81.5%)
3	7	(20.6%)	2	(9.5%)	6	(21.4%)	3	(11.1%)
**Stage**								
I	8	(23.5%)	7	(33.3%)	8	(28.6%)	7	(25.9%)
II	2	(5.9%)	4	(19.0%)	3	(10.7%)	3	(11.1%)
III	6	(17.6%)	5	(23.8%)	7	(25.0%)	4	(14.8%)
IV	18	(52.9%)	5	(23.8%)	10	(35.7%)	13	(48.1%)

Table S4. Patient characteristics according to dichotomized CD163+ tumor center and CD163+ invasion front cell counts.

	CD163+ tumor center				CD163+ invasion front			
	low		high		low		high	
Variables/Categories	n	(%)	n	(%)	n	(%)	n	(%)
**Number of patients**	33	(100.0%)	22	(100.0%)	25	(100.0%)	30	(100.0%)
**Age**								
≥70	11	(33.3%)	9	(40.9%)	9	(36.0%)	11	(36.7%)
<70	22	(66.7%)	13	(59.1%)	16	(64.0%)	19	(63.3%)
**Gender**								
male	24	(72.7%)	15	(68.2%)	19	(76.0%)	20	(66.7%)
female	9	(27.3%)	7	(31.8%)	6	(24.0%)	10	(33.3%)
**Tumor spread**								
1	12	(36.4%)	8	(36.4%)	9	(36.0%)	11	(36.7%)
2	8	(24.2%)	7	(31.8%)	8	(32.0%)	7	(23.3%)
3	4	(12.1%)	3	(13.6%)	2	(8.0%)	5	(16.7%)
4	9	(27.3%)	4	(18.2%)	6	(24.0%)	7	(23.3%)
**Nodal metastasis**								
0	19	(57.6%)	11	(50.0%)	10	(40.0%)	20	(66.7%)
+	14	(42.4%)	11	(50.0%)	15	(60.0%)	10	(33.3%)
**Metastasis**								
no spread	25	(75.8%)	15	(68.2%)	19	(76.0%)	21	(70.0%)
any spread	4	(12.1%)	3	(13.6%)	4	(16.0%)	3	(10.0%)
unknown	4	(12.1%)	4	(18.2%)	2	(8.0%)	6	(20.0%)
**Grade**								
1	0	(0.0%)	2	(9.1%)	0	(0.0%)	2	(6.7%)
2	28	(84.8%)	16	(72.7%)	21	(84.0%)	23	(76.7%)
3	5	(15.2%)	4	(18.2%)	4	(16.0%)	5	(16.7%)
**Stage**								
I	10	(30.3%)	5	(22.7%)	6	(24.0%)	9	(30.0%)
II	3	(9.1%)	3	(13.6%)	1	(4.0%)	5	(16.7%)
III	5	(15.2%)	6	(27.3%)	6	(24.0%)	5	(16.7%)
IV	15	(45.5%)	8	(36.4%)	12	(48.0%)	11	(36.7%)

Table S5. Patient characteristics according to dichotomized M1 tumor center and M1 invasion front cell counts.

	M1 tumor center				M1 invasion front			
	low		high		low		high	
Variables/Categories	n	(%)	n	(%)	n	(%)	n	(%)
**Number of patients**	38	(100.0%)	17	(100.0%)	35	(100.0%)	20	(100.0%)
**Age**								
≥70	12	(31.6%)	8	(47.1%)	13	(37.1%)	7	(35.0%)
<70	26	(68.4%)	9	(52.9%)	22	(62.9%)	13	(65.0%)
**Gender**								
male	30	(78.9%)	9	(52.9%)	23	(65.7%)	16	(80.0%)
female	8	(21.1%)	8	(47.1%)	12	(34.3%)	4	(20.0%)
**Tumor spread**								
1	12	(31.6%)	8	(47.1%)	14	(40.0%)	6	(30.0%)
2	11	(28.9%)	4	(23.5%)	9	(25.7%)	6	(30.0%)
3	5	(13.2%)	2	(11.8%)	4	(11.4%)	3	(15.0%)
4	10	(26.3%)	3	(17.6%)	8	(22.9%)	5	(25.0%)
**Nodal metastasis**								
0	19	(50.0%)	11	(64.7%)	18	(51.4%)	12	(60.0%)
+	19	(50.0%)	6	(35.3%)	17	(48.6%)	8	(40.0%)
**Metastasis**								
no spread	28	(73.7%)	12	(70.6%)	25	(71.4%)	15	(75.0%)
any spread	6	(15.8%)	1	(5.9%)	6	(17.1%)	1	(5.0%)
unknown	4	(10.5%)	4	(23.5%)	4	(11.4%)	4	(20.0%)
**Grade**								
1	0	(0.0%)	2	(11.8%)	1	(2.9%)	1	(5.0%)
2	31	(81.6%)	13	(76.5%)	28	(80.0%)	16	(80.0%)
3	7	(18.4%)	2	(11.8%)	6	(17.1%)	3	(15.0%)
**Stage**								
I	9	(23.7%)	6	(35.3%)	9	(25.7%)	6	(30.0%)
II	3	(7.9%)	3	(17.6%)	5	(14.3%)	1	(5.0%)
III	7	(18.4%)	4	(23.5%)	6	(17.1%)	5	(25.0%)
IV	19	(50.0%)	4	(23.5%)	15	(42.9%)	8	(40.0%)
